# Hypoxemia during rapid eye movement sleep mediates memory impairment in older adults at risk for dementia via CA1 hippocampal volume loss

**DOI:** 10.1111/ene.16491

**Published:** 2024-09-20

**Authors:** Aaron Lam, Angela L. D’Rozario, Jake R. Palmer, Andrew C. McKinnon, Marshall A. Dalton, Nicole Espinosa, Loren Mowszowski, Craig L. Phillips, Ronald R. Grunstein, Sharon L. Naismith

**Affiliations:** ^1^ Healthy Brain Ageing Program, Brain and Mind Centre University of Sydney Sydney New South Wales Australia; ^2^ Faculty of Science, School of Psychology University of Sydney Sydney New South Wales Australia; ^3^ Centre for Sleep and Chronobiology Woolcock Institute of Medical Research, Macquarie University Sydney New South Wales Australia; ^4^ Faculty of Medicine, Health, and Human Sciences, School of Psychological Sciences Macquarie University Sydney New South Wales Australia; ^5^ Charles Perkins Centre University of Sydney Sydney New South Wales Australia; ^6^ CogSleep Centre of Research Excellence, National Health and Medical Research Council Sydney New South Wales Australia; ^7^ Royal Prince Alfred Hospital Sydney New South Wales Australia; ^8^ Faculty of Medicine, Health and Human Sciences, Macquarie Medical School Macquarie University Sydney New South Wales Australia

**Keywords:** ageing, cognition, dementia, neuroimaging, obstructive sleep apnea

## Abstract

**Background and Purpose:**

Obstructive sleep apnea is associated with increased dementia risk. Nocturnal hypoxemia, which can be more severe during rapid eye movement (REM) sleep, may be a key mechanism. This study examines how REM hypoxemia affects memory and explores whether hippocampal vulnerability to hypoxemia mediates this effect in older adults at risk for dementia.

**Methods:**

Older adults aged ≥50 years (*N* = 338) with subjective or mild cognitive impairment (i.e., objective impairment) underwent neuropsychological, mood, and medical assessment, magnetic resonance imaging scanning (*n* = 135), and overnight polysomnography. Verbal learning and memory were assessed with the Rey Auditory Verbal Learning Test. REM sleep hypoxemia was measured using the Oxygen Desaturation Index‐3% (REM‐ODI). Hippocampal subfield (CA1, CA3, subiculum, and dentate gyrus) volumes were derived from T1 and high‐resolution hippocampus T2 scans. We determined whether the relationship between REM‐ODI and learning and memory was mediated by hippocampal subfield volume. Analyses were repeated in non‐REM sleep to determine whether the effects were REM‐specific.

**Results:**

Although there was not a direct effect of REM‐ODI on verbal learning (*p* > 0.05) or memory (*p* > 0.05), mediation analyses showed a significant indirect effect of high REM‐ODI on poorer verbal learning (*β* = −0.09, 95% confidence interval [CI] = −0.238 to −0.005) and memory (*β* = −0.100, 95% CI = −0.255 to −0.005), which was mediated by CA1 volume. These associations were absent in non‐REM sleep (*p* > 0.05).

**Conclusions:**

Hypoxemia during REM sleep may impair memory in people at risk for dementia by reducing CA1 hippocampal volume. Research is needed to explore whether interventions targeting REM sleep hypoxemia are protective against memory decline.

## INTRODUCTION

Obstructive sleep apnea (OSA) is characterized by repetitive periods of cessation and/or reduction of airflow during sleep, leading to intermittent hypoxemia (i.e., repeated drops in blood oxygen levels). The prevalence rates of OSA are up to 70% in older adults [1], a concerning statistic given the association of OSA with cognitive decline [2] and dementia [3]. The links between OSA and cognition warrant closer inspection in older adults with concomitant cognitive concerns [4], because this at‐risk group provides a critical opportunity for secondary dementia prevention strategies [5].

Individuals considered to be at risk for dementia include those with subjective cognitive impairment (SCI), who report cognitive concerns but do not yet show objective cognitive deficits, and those with mild cognitive impairment (MCI), who display cognitive deficits on neuropsychological testing. Those with amnestic MCI (aMCI; i.e., predominant memory deficits) are at the greatest risk of conversion to dementia, particularly Alzheimer disease (AD) [6], with approximately 45% converting to dementia over 5 years [6]. Previous research showed that despite presenting with cognitive concerns, individuals with SCI or MCI exhibit moderate levels of OSA. Notably, intermittent hypoxemia due to OSA is linked with temporal lobe brain atrophy, poor learning performance [7], impaired sleep‐dependent memory [8], and functional uncoupling within the brain's default mode network, specifically in the bilateral parahippocampal cortex [9].

The hippocampus, which plays a crucial role in episodic memory, is known to be affected early in AD [10]. Recent human imaging studies have further delineated the specific roles of various hippocampal subfields in episodic memory functions [11]. Poorer verbal memory has been associated with decreased subfield volumes, including the CA1 [12] and subiculum [13] in healthy older adults, and both the CA1 and dentate gyrus (DG) in aMCI [11]. This reduction in hippocampal volume may suggest atrophy and neuronal loss, but could also reflect reduced tissue water content [14] and reduced synaptic spine density and dendritic volume [15]. Animal models focused on hypoxemia have shown that the CA1 and DG subfields are particularly susceptible to hypoxic injury [16]. Moreover, human imaging and postmortem studies of typical OSA samples show damage to both the CA1 and CA3 subfields [17]. However, the interrelationships between hippocampal subfields, hypoxemia, and memory are unknown, particularly in older adults at risk for dementia.

An important consideration in both OSA and the ageing field is the role of rapid eye movement (REM) sleep. REM sleep is important for long‐term depotentiation and neuroplasticity [18], and alterations in REM sleep neurophysiology have been observed in individuals at risk for dementia [19]. Among older adults, reduced REM sleep duration and increased REM sleep latency are associated with a heightened risk of AD [20, 21]. During REM sleep, there is a further decrease in genioglossus muscle tone, leading to a greater tendency to have obstructive events [22]. These events, due to a decreased respiratory drive, are associated with greater oxygen desaturation [23]. Consequently, it is plausible that hypoxemia during REM sleep may preferentially contribute to hippocampal damage and memory decrements.

This study aimed to determine whether hypoxemia during REM sleep was linked to poorer verbal learning and memory performance in older adults at risk for dementia and to explore whether this relationship was mediated by hippocampal subfield volume loss. Based on prior research, we selected four hippocampal subfields (CA1, CA3, DG, and subiculum) for investigation, as they are known to be linked with memory and were previously shown to be affected by OSA or hypoxemia in either humans or animal studies. Finally, to determine whether these relationships were specific to REM sleep, the analyses were repeated for non‐REM (NREM) sleep stages. We hypothesized that hypoxemia during REM sleep would be linked to poorer verbal learning and memory performance, and this would be mediated by reduced hippocampal subfield volumes, specifically the CA1, CA3, DG, and subiculum. Furthermore, given hypoxemia is more severe during REM sleep, we hypothesized that this effect would not be observed during NREM sleep.

## MATERIALS AND METHODS

Participants were recruited from the Healthy Brain Ageing Clinic at the Brain and Mind Centre, University of Sydney, Australia. Participants were referred by their primary care or specialist physician for a detailed assessment of early cognitive concerns.

The inclusion criteria for this study were as follows: participants had to be aged 50 years or older, have a Mini‐Mental State Examination (MMSE) [24] score of >23, possess sufficient English proficiency for standardized neuropsychological assessment, and have undergone an overnight polysomnography (PSG) within 6 months of the clinical assessment.

The exclusion criteria were as follows: a diagnosis of dementia, history of stroke and/or traumatic brain injury with a loss of consciousness for >30 min, any neurological disease (e.g., epilepsy, Parkinson disease), a history of significant substance misuse, a major psychiatric disorder (e.g., schizophrenia or bipolar disorder), having slept <4 h on the night of their PSG, and being a current continuous positive airway pressure (CPAP) user.

### Protocol approvals, registrations, and patient consent

This study was approved by the University of Sydney Human Research Ethics Committee (Protocol No. 02–2011/13515). Written informed consent was obtained from all participants prior to any study procedures.

### Clinical assessment

As described previously [25], the assessment included the following:

- *Medical assessment*: A geriatrician or neurologist obtained a medical history, body mass index, physical examination, current medication, and supplement use. The use of antihypertensive, cholesterol‐lowering, or antidepressant medication, and weekly alcohol consumption were recorded. The Cumulative Illness Rating Scale–Geriatric version (CIRS‐G) was administered as a measure of medical burden [26].
- *Neuropsychological assessment*: A clinical neuropsychologist conducted a standardized test battery suitable for the detection of early cognitive decline and dementia. The MMSE (a measure of global cognition) [24] and the Wechsler Test of Adult Reading (to estimate premorbid intelligence) [27] were reported for descriptive purposes. The primary outcomes of this study were verbal learning and memory using the Rey Auditory Verbal Learning Test [28]. The participants were asked to learn and encode a 15‐item word list across five learning trials. The age and education normative score [29] was used to assess *verbal learning*. *Verbal memory* was tested following a 20‐min delay.
- *Psychologist assessment*: A research psychologist conducted a semistructured interview covering sleep history and the Mini International Neuropsychiatric Interview [30] for affective disorder diagnoses.
- *Self‐report measures*: These included the 15‐item Geriatric Depression Scale [31] to measure depressive symptoms in older adults and the Pittsburgh Sleep Quality Index [32] as a measure of subjective sleep quality.

### Clinical SCI and MCI classifications

The classifications of SCI, non‐amnestic MCI (naMCI), and aMCI were consensus‐rated by a geriatrician and at least one clinical neuropsychologist. The MCI criteria required deficits of at least 1.5 SD below premorbid estimates (following Winblad criteria) [33]. The subtypes of aMCI and naMCI were defined according to the presence or absence of memory deficits, respectively, and rated according to whether single or multiple domains of cognition were affected. SCI was classified when MCI was not evident on neuropsychological testing, but subjective cognitive impairments were present.

### Magnetic resonance imaging

A subset of participants (*n* = 135) completed the magnetic resonance imaging (MRI) protocol at the Brain and Mind Centre, Sydney, within 59 days of clinical assessment. Prior to July 2019, the structural MRI data were collected using a 3‐T General Electric (GE) Discovery MR750 scanner (GE Medical Systems, Chicago, IL, USA) with an eight‐channel phased‐array head coil. The following T1‐weighted imaging sequence was used: three‐dimensional (3D) T1‐weighted brain volume imaging (BRAVO) spoiled gradient‐recalled sequence (phase acceleration factor = 2), acquiring 196 sagittal slices (repetition time = 7.2 ms, echo time = 2.8 ms, flip angle = 12, matrix 256 × 256, 0.9‐mm isotropic voxels), acquisition time = 4 min, 27 s. Furthermore, the T2 high‐resolution hippocampus sequence was the following: 2D T2‐weighted fast spin echo‐extended length (FSE‐XL) sequence, acquiring 24 sagittal slices (repetition time = 3000.0 ms; echo time = 51.48 ms; flip angle = 111; matrix 512 × 512; 0.25‐, 0.25‐, 2.0‐mm isotropic voxels), acquisition time = 6 min, 12 s. For those scans acquired after July 2019, the structural MRI data were acquired using a 32‐channel phased‐array head coil. The following changes to the T1‐weighted imaging sequence were implemented: repetition time = 7.4 ms; echo time = 3.0 ms; flip angle = 11; matrix 256 × 256; 1.0‐mm isotropic voxels; and acquisition time = 4 min, 5 s. No changes were made to the T2 high‐resolution hippocampus sequence.

### Hippocampal volumes

For hippocampal segmentation analysis, we applied automated segmentation of hippocampal subfields software with both 3D T1‐weighted and high‐resolution T2‐weighted images (https://sites.google.com/view/ashs‐dox/home) [34]. We used the Penn Memory Center T2‐Weighted 3T MRI Atlas as the Atlas set [34]. This software automatically calculated the volumes of each subfield with a combination of learning‐based error correction and multiatlas label fusion. Four regions of interest were delineated: CA1, CA3, DG, and subiculum. Each subfield was corrected for individual estimated intracranial volume by converting the subfield volume to the percentage of total intracranial volume ([absolute subfield volume / estimated intracranial volume] * 100). All images were visually inspected for quality control, and only those hippocampal subfields with segmentation errors were excluded for each participant.

### Overnight PSG

All sleep studies were completed within 155 days of the neuropsychological assessment. Overnight PSG was conducted at the Woolcock Institute of Medical Research, Sydney and recorded using an Alice‐3 (Healthdyne Technologies, Florida, USA), Compumedics Siesta (Compumedics Limited, Victoria, Australia), Embla Titanium (Mortara Instruments, Colorado, USA), or Sandman Elite (Tyco Healthcare, Connecticut, USA) system. Sleep architecture and respiratory events were manually scored by a trained sleep technician according to American Academy of Sleep Medicine v2.2 guidelines [35]. The primary outcome, REM–oxygen desaturation index (ODI; number of oxygen desaturations events ≥3% during REM sleep), was calculated from finger pulse oximetry recorded during PSG. For descriptive purposes, the following were also reported: total sleep time, time spent in NREM sleep, time spent in REM sleep, wake after sleep onset, sleep efficiency, apnea–hypopnea index, ODI during NREM sleep, time spent below 90% oxygen saturation (SpO2), and minimum SpO2 during NREM and REM sleep.

### Statistical analyses

All statistical analyses were performed using the Statistical Package for Social Sciences (SPSS version 24) [36]. Power analyses were conducted using G*Power (version 3.1). All outcome variables were inspected for normality using skewness, kurtosis, and Shapiro–Wilk tests. Log transformations were performed on nonnormal distributions; however, for variables that were still nonnormal after transformation, nonparametric analyses were performed in place of parametric tests. Pearson correlation was used to examine the relationship between REM‐ODI and verbal learning and memory.

To assess the role of hippocampal subfields as a mediator in the relationship between REM‐ODI and verbal learning and memory, mediation analysis (model 4) was performed using PROCESS version 4.2 for SPSS [37]. The mediation analyses were informed by two pathways: step 1, association between REM‐ODI and hippocampal subfield; step 2, association of hippocampal subfield and verbal learning or memory. If these two pathways were significant, then mediation analyses were conducted. Pathways were tested using Pearson correlation. Both Pearson correlation and mediation analyses were conducted while adjusting for different head coils (eight‐ or 32‐channel coils) and sex. An a priori power calculation was conducted to determine the necessary sample size for detecting the mediation effect in our model. A sample size of 86 was necessary to detect a medium effect size (*f* ^2^ = 0.15, power = 0.80, alpha level = 0.05) for path c, the direct effect of the independent variable on the dependent variable while controlling for the mediation variable. For paths a and b, a sample size of 55 was necessary to detect a medium effect size (*f* ^2^ = 0.15, power = 0.80, alpha level = 0.05).

To ensure that associations between ODI and memory were specific to REM sleep, analyses were repeated for ODI during NREM sleep. This process involved replicating the correlation analyses to guide the subsequent mediation analyses for ODI during NREM sleep.

## RESULTS

### Demographics and clinical characteristics

Three hundred thirty‐eight participants were included in this analysis, comprising 109 participants with SCI and 229 with MCI. Table 1 shows the demographic and clinical characteristics of the sample (and is further broken down by SCI and MCI in Table S1). Briefly, participants with MCI were, on average, older, had higher medical illness burden (CIRS‐G), and consumed less alcohol weekly compared to participants with SCI. No other differences, including sleep measures, were observed between groups.

**TABLE 1:** Whole sample demographics and clinical characteristics (*N* = 338).

Characteristic	Mean ± SD
Age, years	67.0 ± 8.2
Education, years	14.3 ± 3.0
Sex, male, *n* (%)	139 (41%)
Body mass index	27.3 ± 5.5
Mini‐Mental State Examination, /30	28.7 ± 2.0
Geriatric Depression Scale–15 items, /15	3.9 ± 3.5
Cumulative Illness Rating Scale–Geriatric version, /52	44.9 ± 9.4
Alcohol use, drinks/week	5.2 ± 3.4
Pittsburgh Sleep Quality Index, /21	7.3 ± 3.7
Total sleep time, min	343.8 ± 62.8
Wake after sleep onset, min	80.3 ± 50.3
Sleep efficiency, %	75.1 ± 13.5
Sleep latency, min	30.8 ± 39.8
Duration of NREM, min	283.9 ± 52.2
Duration of REM, min	59.9 ± 28.0
Apnea–hypopnea index for total sleep time, events/h	16.4 ± 16.4

Abbreviations: NREM, non‐REM; REM, rapid eye movement.

A subset of 135 participants completed the necessary MRI sequences. Table S2 presents group differences in demographic and clinical measures between participants who completed the MRI (*n* = 135) and those who did not (*n* = 203). On average, participants who completed the MRI had higher weekly alcohol intake, better self‐reported sleep quality, more NREM sleep duration, lower ODI during total sleep and REM sleep, and higher minimum SpO [2] during both NREM and REM sleep.

The median time from the neuropsychological assessment to the PSG was 23 days (range = 0–155 days). The median length of time from the MRI scan to the overnight PSG was 11 days (range = 0–63 days). The median time from the neuropsychological assessment to the MRI scan was 15 days (range = 0–59 days).

### Associations between verbal learning and memory and ODI

To explore the relationship between ODI and memory, we conducted Pearson correlations. In the entire sample (*N* = 338), ODI during REM or NREM sleep was not significantly associated with either verbal learning or memory. See Table 2 for the full correlation matrix.

**TABLE 2:** Correlational analyses between learning and memory and oxygen desaturation indices during total sleep time, REM sleep, and NREM sleep (*N* = 338).

	Total ODI	REM ODI	NREM ODI
Verbal learning			
Pearson *r*	−0.044	−0.017	−0.050
*p*	0.401	0.744	0.344
Verbal memory			
Pearson *r*	−0.044	−0.019	−0.054
*p*	0.399	0.721	0.301

Abbreviations: NREM, non‐REM; ODI, oxygen desaturation index; REM, rapid eye movement.

### Associations between ODI and hippocampal subfields

Pearson partial correlation coefficient (adjusting for sex and different MRI sequences) showed that REM‐ODI was negatively correlated with CA1 volume (*r* = −0.303, *p* < 0.05). There were no other significant correlations between ODI during REM or NREM sleep and hippocampal subfield volumes. The full correlation matrix is presented in Table S3.

### Associations between hippocampal subfields and verbal learning and memory

Pearson partial correlation coefficient (adjusting for sex and different MRI sequences) revealed that both poorer verbal learning and poorer memory were associated with lower CA1 and DG volumes (see full correlation matrix in Table 3). There were no other significant associations between verbal learning or memory and hippocampal subfield volumes.

**TABLE 3:** Correlational analyses between hippocampal subfields and learning and memory performance.

	Verbal learning	Verbal memory
CA1, *n* = 55		
Pearson *r*	0.349**	0.337*
*p*	0.008	0.010
CA3, *n* = 118		
Pearson *r*	−0.041	−0.058
*p*	0.660	0.527
Dentate gyrus, *n* = 87		
Pearson *r*	0.239*	0.235*
*p*	0.024	0.026
Subiculum, *n* = 98		
Pearson *r*	0.039	0.039
*p*	0.703	0.703

*Note*: Data are presented as partial Pearson correlation coefficient, adjusting for sex and different magnetic resonance imaging sequences.

* *p* < 0.05.

** *p* < 0.01.

### Mediation analyses

**FIGURE 1:**
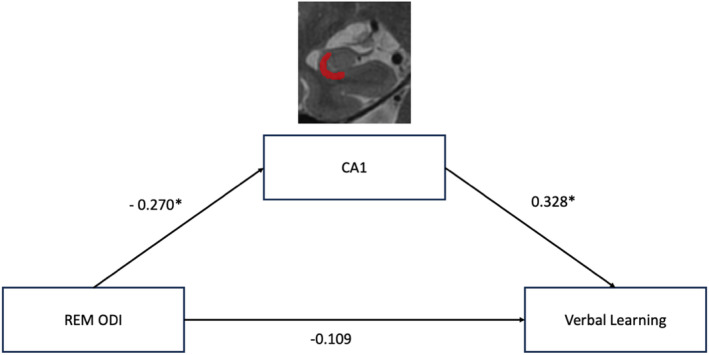
Model of the mediating analysis of CA1 on the association between rapid eye movement (REM)–oxygen desaturation index (ODI) and verbal learning. The figure depicts the mediating effects of CA1 on the relationship between REM‐ODI and verbal learning performance. The model includes direct and indirect paths with corresponding path coefficients. The indirect effect through CA1 was significant (*β* = −0.088, *p* < 0.05). There was no significant effect of REM‐ODI on verbal learning (*β* = −0.109, *p* > 0.05). Indirect effect of REM‐ODI on verbal learning = −0.088, 95% confidence interval = −0.238 to −0.005. Significant paths were indicated by *.

**FIGURE 2:**
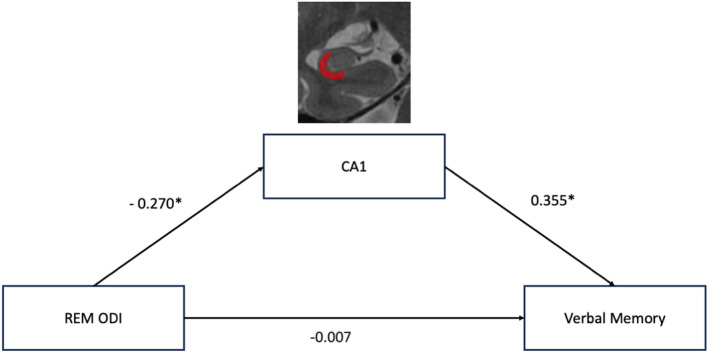
Model of the mediating analysis of CA1 on the association between rapid eye movement (REM)–oxygen desaturation index (ODI) and verbal memory. The figure depicts the mediating effects of CA1 on the relationship between REM‐ODI and verbal memory performance. The model includes direct and indirect paths with corresponding path coefficients. The indirect effect through CA1 was significant (*β* = −0.096, *p* < 0.05). There was no significant effect of REM‐ODI on verbal memory (*β* = −0.007, *p* > 0.05). Indirect effect of REM‐ODI on verbal memory = −0.096, 95% confidence interval = −0.255 to −0.005. Significant paths were indicated by *.

A subset of 55 participants who met visual quality checks were included in the CA1 mediation analysis. After adjusting for sex and MRI head coil differences, there was no direct effect of REM‐ODI on either verbal learning (standardized beta = −0.109, *p* > 0.05) or memory (standardized beta = −0.007, *p* > 0.05). However, the relationship between REM‐ODI and CA1 volume was significant (standardized beta = −0.270, *p* < 0.05), suggesting that higher REM‐ODI was linked to lower CA1 volume. The association of CA1 with both verbal learning (standardized beta = 0.328, *p* < 0.05) and memory (standardized beta = 0.355, *p* < 0.05) was significant, suggesting that smaller CA1 volume was related to poorer verbal learning and memory performance. Importantly, the mediation analysis demonstrated a significant indirect effect of REM‐ODI on verbal learning (standardized beta = −0.09, 95% confidence interval [CI] = −0.238 to −0.005) and memory (standardized beta = −0.100, 95% CI = −0.255 to −0.005) through CA1 volume. Because there were no significant correlations between NREM sleep and hippocampal subfields, no mediation analyses were conducted for NREM sleep. Learning and memory full mediation models are presented in Figures 1 and 2, respectively.

## DISCUSSION

This study examined the relationship between intermittent hypoxemia during REM sleep and verbal episodic learning and memory performance in older individuals at risk for dementia. Our findings partially supported our initial hypotheses. Although REM hypoxemia was not significantly associated with verbal learning and memory, it was linked to decreased CA1 volume. Furthermore, REM hypoxemia mediated poorer verbal learning and memory via decreased CA1 hippocampal volume. Furthermore, we demonstrated that this relationship was specific to REM (not NREM) sleep.

Although the effect size was modest, our findings pertaining to REM‐ODI and memory provide valuable new insights into the pathways linking OSA and memory in older adults. In particular, they may be relevant for clinical practice and for informing future studies that focus on ODI during REM sleep specifically, as opposed to ODI throughout the night. Our findings align with a recently published study suggesting that REM hypoxemia, rather than NREM, was more critical for memory impairment in 81 cognitively intact older adults [38]. Our study expands on this by including individuals with subjective and objective cognitive impairment, broadening the understanding of REM hypoxemia's role in memory systems across the cognitive decline continuum. Additionally, we examined the role of REM‐ODI in the neurodegeneration of brain regions critical for memory, namely the hippocampus. Given that this association is indirect, it is a crucial factor to consider when exploring the links between OSA and cognitive decline. This may also explain some of the variability in the literature regarding the relationship between OSA and memory. That is, perhaps clinically significant memory decrements only manifest once a sufficient degree of hippocampal compromise has occurred. Future studies may examine this relationship more closely and consider the interrelationship between REM hypoxemia and other biomarkers of neurodegeneration. In this regard, prior studies have shown that hypoxemia is linked with neuroinflammation, oxidative stress, and amyloid and tau deposition [39] and hence, may shorten the timeline for the progression to AD.

Moreover, the findings that showed unique effects during REM sleep, which were not observed in NREM sleep, could be explained by multiple non‐mutually exclusive factors. First, previous studies have shown that during REM sleep, OSA can be more severe with greater drops in oxygen saturation and longer duration events [40]. Consequently, hypoxemia events during REM sleep may be particularly deleterious relative to those during NREM sleep. Second, the brain is more vulnerable during REM sleep due to the physiological features of the sleep state [41]. Baril and colleagues [42] demonstrated that mild REM sleep OSA was associated with impaired daytime cerebral perfusion, an association that was not present for mild NREM sleep OSA. Third, neurovascular coupling is dependent on NREM and REM sleep states, with delayed blood volume responses occurring during REM sleep [43]. Subsequently, during REM sleep, the brain is more susceptible to hypoxemia, as reoxygenation may be delayed. Therefore, a combination of more severe hypoxemia events and a vulnerable brain state during REM sleep may contribute to a greater risk of neurodegeneration and subsequent cognitive decline.

This study has important implications for understanding the pathological links between OSA, cognition, and dementia as well as for clinical practice, particularly in clinical samples at risk for dementia. Based on the current findings and prior work [7, 9], implementation of OSA screening in the memory clinic setting appears warranted, using either in‐laboratory PSG or home pulse oximetry. Conversely, those presenting to sleep clinics with high REM‐ODI could undergo formal neuropsychological examination assessing cognition generally and episodic memory specifically [5]. Most REM sleep occurs in the latter stages of the night, and therefore hypoxemia during REM sleep will be untreated if there is premature (midsleep) cessation of CPAP treatment. The findings also suggest that future work in this field should consider examining ODI in REM versus NREM sleep in older clinical samples.

The strengths of this study lie in its well‐phenotyped clinical sample with comprehensive medical, neuropsychological, psychological, and gold‐standard sleep assessments, as well as the specific examination of the hippocampus. The hippocampus is a key structure that supports episodic memory and is affected early in the progression of AD. Several limitations are worth noting, including the use of an automated segmentation methodology for hippocampal subfields with a strict visual quality check. This methodology led to the exclusion of various scans and subfield volumes due to poor segmentation. Another limitation of our study was that we were unable to utilize the National Institute on Aging and Alzheimer's Association Research Framework [44] to characterize our participants according to AD biomarkers and to incorporate AD biomarkers based on positron emission tomography, cerebrospinal fluid, or plasma. Therefore, we could not capture the biological heterogeneity and underlying causes of cognitive decline within our sample. It is noted that our sample included people with concerns about their memory and therefore has a restricted range. That is, there is less representation of those who are cognitively intact or with moderate to severe dementia. It is thus prudent to interpret these results in light of the sample examined. Furthermore, although the ODI captures the number of desaturation episodes and is commonly used as an indicator of OSA severity, it does not fully characterize the depth of each desaturation event. For example, the ODI treats a 30‐s episode with a 5% desaturation as equivalent to a 12‐s episode with a 3% desaturation. Consequently, future studies should consider investigating other measures of hypoxemia that can capture the depth of each event, such as the hypoxic burden index. Finally, given the cross‐sectional nature of this study, causality cannot be established. Future follow‐up studies with our sample will be crucial to determine the prognostic relevance of REM‐ODI for the longitudinal progression of memory decline.

The findings show that more intermittent hypoxemia during REM sleep is associated with poorer learning and memory via decreased hippocampal CA1 volume. The absence of these findings in NREM sleep supports the specificity of this relationship with REM sleep. Clinically, our results highlight the importance of screening for OSA and REM‐ODI, particularly if effective treatment can mitigate these issues. However, more evidence is necessary to determine the efficacy of sleep apnea treatment.

## AUTHOR CONTRIBUTIONS

**Aaron Lam:** Conceptualization; investigation; writing – original draft; writing – review and editing; formal analysis; methodology; visualization; project administration. **Angela L. D'Rozario:** Writing – review and editing; conceptualization; supervision; methodology; project administration. **Jake R. Palmer:** Writing – review and editing; data curation. **Andrew C. McKinnon:** Writing – review and editing; data curation; formal analysis. **Marshall A. Dalton:** Writing – review and editing; methodology. **Nicole Espinosa:** Visualization; data curation; writing – review and editing; validation; project administration. **Loren Mowszowski:** Writing – review and editing; methodology; project administration. **Craig L. Phillips:** Writing – review and editing; supervision. **Ronald R. Grunstein:** Writing – review and editing; supervision; conceptualization; methodology; project administration. **Sharon L. Naismith:** Funding acquisition; conceptualization; investigation; writing – review and editing; supervision; methodology; writing – original draft; project administration.

## CONFLICT OF INTEREST STATEMENT

S.L.N. has received consulting fees for being on the Lecanemab advisory board from Eisai Australia, as well as speaker fees from Roche and Nutricia. R.R.G. has received research funding from Eli Lilly to undertake the Surmount‐OSA and Triumph clinical trials. There are no other conflicts of interest to declare.

## Supporting information


Data S1.

## Data Availability

The data supporting this study's findings are available from the corresponding author upon reasonable request.
