# Supplementary material for: Hypoxemia during rapid eye movement sleep mediates memory impairment in older adults at risk for dementia via CA1 hippocampal volume loss
Source: Eur J Neurol. 2024 Sep 20;31(12):e16491. doi: 10.1111/ene.16491 (PMC11555004; doi:10.1111/ene.16491)
Supplement: Supplementary file 1 — Data S1. [file ENE-31-e16491-s001.docx]

Supplementary Materials

| *Supplementary Table 1. Comparison between participants with SCI and MCI* | | | | |
| --- | --- | --- | --- | --- |
|  | SCI  n=109 | MCI  n=229 | Test Statistic | p-value |
| Age, years | 64.5 ± 8.0 | 68.0 ± 8.0 | -3.814*** | 0.001 |
| Education, years | 14.2 ± 2.7 | 14.4 ± 3.1 | -0.436 | 0.663 |
| Sex, male, n (%)^a^ | 39 (36%) | 100 (44%) | 1.898 | 0.168 |
| Body Mass Index | 27.5 ± 6.1 | 27.3 ± 5.5 | 0.323 | 0.747 |
| Mini-Mental State Examination, /30 | 29.1 ± 2.3 | 28.5 ± 1.9 | 2.529* | 0.012 |
| Geriatric Depression Scale-15 items, /15 | 4.3 ± 3.6 | 3.6 ± 3.4 | 1.587 | 0.114 |
| Cumulative Illness Rating Scale-Geriatric, /52 | 4.5 ± 2.8 | 5.6 ± 3.7 | -2.789*** | 0.006 |
| Alcohol use (drinks/wk) | 6.5 ± 7.7 | 4.5 ± 6.5 | 2.401* | 0.017 |
| Pittsburgh Sleep Quality Index, /21 | 7.7 ± 3.8 | 7.1 ± 3.6 | 1.106 | 0.270 |
| Total sleep time, mins | 350.1 ± 60.4 | 338.2 ± 64.6 | 1.623 | 0.105 |
| Wake after sleep onset, mins | 77.5 ± 45.7 | 83.6 ± 54.1 | -1.019 | 0.309 |
| Sleep efficiency % | 76.1 ± 12.7 | 74.1 ± 14.2 | 1.259 | 0.209 |
| Sleep latency, mins | 31.1 ± 42.2 | 31.9 ± 40.7 | -0.169 | 0.866 |
| Duration of NREM, mins | 288.3 ± 51.2 | 281.5 ± 54.0 | 1.097 | 0.273 |
| Duration of REM, mins | 61.8 ± 25.1 | 56.6 ± 28.5 | 1.619 | 0.106 |
| Apnea-hypopnea index for total sleep time (p/h) | 16.4 ± 18.2 | 16.8 ± 15.9 | -0.204 | 0.838 |
| Oxygen desaturation index for total sleep time (p/h) | 10.2 ± 12.8 | 10.6 ± 12.1 | -0.263 | 0.793 |
| Oxygen desaturation index during NREM sleep (p/h) | 8.9 ± 12.8 | 9.4 ± 12.0 | -0.349 | 0.727 |
| Oxygen desaturation index during REM sleep (p/h) | 16.7 ± 18.9 | 17.0 ± 17.8 | -0.117 | 0.907 |
| Time spent < 90% SpO2 (minutes) | 2.1 ± 6.1 | 3.4 ± 8.6 | -1.150 | 0.251 |
| Minimum SpO2 during NREM (%) | 88.3 ± 4.9 | 87.0 ± 6.6 | 1.868 | 0.063 |
| Minimum SpO2 during REM (%) | 88.1 ± 6.4 | 87.6 ± 7.0 | 0.588 | 0.557 |
| *p<0.05, **p<0.01, ***p<0.001. Mean and standard deviations are presented unless stated otherwise. All tests conducted are independent samples t-test unless stated otherwise. ^a^ Chi-square goodness of fit was run. | | | | |

| *Supplementary Table 2. Comparison between participants with and without MRI* | | | | |
| --- | --- | --- | --- | --- |
|  | Without MRI  n=203 | With MRI  n=135 | Test Statistic | p-value |
| Age, years | 67.2 ± 8.2 | 66.4 ± 8.2 | 0.862 | 0.389 |
| Education, years | 14.2 ± 3.1 | 14.4 ± 2.8 | -0.554 | 0.580 |
| Sex, male, n (%)^a^ | 80 (39%) | 59 (44%) | 0.618 | 0.432 |
| Body Mass Index | 27.8 ± 5.8 | 26.6 ± 5.5 | 1.809 | 0.071 |
| Mini-Mental State Examination, /30 | 28.6 ± 2.3 | 28.8 ± 1.6 | -1.184 | 0.069 |
| Geriatric Depression Scale-15 items, /15 | 4.2 ± 3.6 | 3.4 ± 3.3 | 1.946 | 0.053 |
| Cumulative Illness Rating Scale-Geriatric, /52 | 5.5 ± 3.5 | 4.9 ± 3.5 | 1.529 | 0.127 |
| Alcohol use (drinks/wk) | 4.4 ± 5.7 | 6.3 ± 8.4 | -2.451* | 0.015 |
| Pittsburgh Sleep Quality Index, /21 | 8.1 ± 3.9 | 6.4 ± 3.2 | 3.808*** | 0.001 |
| Total sleep time, mins | 337.2 ± 62.6 | 349.3 ± 64.1 | -1.727 | 0.085 |
| Wake after sleep onset, mins | 83.5 ± 53.0 | 78.9 ± 49.4 | 0.793 | 0.428 |
| Sleep efficiency % | 74.3 ± 14.2 | 75.4 ± 13.2 | -0.705 | 0.481 |
| Sleep latency, mins | 33.2 ± 45.4 | 29.3 ± 33.8 | 0.866 | 0.387 |
| Duration of NREM, mins | 278.5 ± 51.3 | 291.6 ± 55.1 | -2.235* | 0.026 |
| Duration of REM, mins | 58.7 ± 29.1 | 57.7 ± 25.0 | 0.319 | 0.750 |
| Apnea-hypopnea index for total sleep time (p/h) | 17.3 ± 16.7 | 15.7 ± 16.7 | 0.845 | 0.398 |
| Oxygen desaturation index for total sleep time (p/h) | 11.7 ± 12.9 | 8.7 ± 11.2 | 2.198* | 0.029 |
| Oxygen desaturation index during NREM sleep (p/h) | 10.3 ± 12.8 | 7.8 ± 11.3 | 1.843 | 0.066 |
| Oxygen desaturation index during REM sleep (p/h) | 18.8 ± 19.0 | 14.1 ± 16.4 | 2.349* | 0.019 |
| Time spent < 90% SpO2 (minutes) | 3.2 ± 8.2 | 2.7 ± 7.6 | 0.472 | 0.637 |
| Minimum SpO2 during NREM (%) | 86.7 ± 6.4 | 88.4 ± 5.4 | -2.591* | 0.010 |
| Minimum SpO2 during REM (%) | 86.9 ± 7.3 | 89.0 ± 5.7 | -2.780*** | 0.006 |
| *p<0.05, **p<0.01, ***p<0.001. Mean and standard deviations are presented unless stated otherwise. All tests conducted are independent samples t-test unless stated otherwise. ^a^ Chi-square goodness of fit was run. | | | | |

| *Supplementary Table 3. Correlational analyses between hippocampal subfields and oxygen desaturation indices during total sleep time, REM sleep and NREM sleep* | | | | |
| --- | --- | --- | --- | --- |
|  |  | Total ODI | REM ODI | NREM ODI |
| CA1 (n = 55) | Pearson’s r | -0.243 | **-0.303*** | -0.210 |
|  | p-value | 0.071 | **0.023** | 0.121 |
| CA3 (n= 117) | Pearson’s r | 0.005 | 0.053 | -0.021 |
|  | p-value | 0.958 | 0.570 | 0.820 |
| Dentate gyrus (n = 86) | Pearson’s r | -0.035 | -0.147 | -0.007 |
|  | p-value | 0.749 | 0.173 | 0.946 |
| Subiculum (n = 97) | Pearson’s r | 0.039 | 0.009 | 0.051 |
|  | p-value | 0.703 | 0.933 | 0.618 |
| *p<0.05, **p<0.01, ***p<0.001. Data is presented as partial Pearson’s correlation coefficient, adjusting for sex, age and different MRI sequences. REM = rapid eye movement; NREM = non-rapid eye movement; ODI = oxygen desaturation index; CA = cornu ammonis. | | | | |
